# Supplementary material for: Are carbon emissions trading and green financial instruments synergistic? -Comprehensive quantitative research based on content analysis
Source: PLoS One. 2024 Mar 7;19(3):e0298601. doi: 10.1371/journal.pone.0298601 (PMC10919601; doi:10.1371/journal.pone.0298601)
Supplement: S1 Data — (ZIP) [file pone.0298601.s001.zip › Data/地级市Z_F工作报告.zip!/╜¡╬≈/╙Ñ╠╢╩╨/└┤╫╘╣1⁄2╓┌║┼í╛┬φ┐╦╩2╛▌═°í┐_2004.docx]

各位代表：

现在，我代表市人民政府向大会作政府工作报告，请予审议，并请各位市政协委员和列席会议的同志提出意见。 一、2003年经济社会发展情况和本届政府工作的简要回顾

过去的一年，是全面建设小康社会的开局年，是鹰潭成立省辖市二十周年的喜庆年，也是本届政府任期的最后一年。一年来，在省委、省政府和中共鹰潭市委的正确领导下，全市上下认真实践“三个代表”重要思想，全面贯彻十六大和十六届三中全会精神，坚持解放思想，与时俱进，同心同德，扎实工作，超额完成了市五届人大五次会议确定的经济和社会发展各项任务，为加快鹰潭崛起奠定了扎实的基础。2003年全市国内生产总值达到7909亿元，比上年增长182％，比年初预期目标高62个百分点，创建市以来新高。其中一产增长40％，二产增长300％，三产增长114％。三次产业的比例为：157∶479∶364。财政总收入达到863亿元，增长279％，其中地方财政收入448亿元，增长239％，分别超过年初预算159和133个百分点。全社会固定资产投资3089亿元，增长554％。社会消费品零售总额2486亿元，增长12％。城镇登记失业率控制在36％以内，人口自然增长率812‰。主要经济指标增幅均高于全省平均水平。

2003年，我们主要抓了以下工作：

(一)加快实施工业化核心战略，效益在结构调整中明显提高

全市规模以上工业企业完成工业增加值同比增长293％，销售收入增长423％，利税总额增长652％，工业效益综合指数11985％。全市工业用电达到774亿千瓦时，同比增长385％。18户重点工业企业发展迅猛。江西铜业集团公司如期完成贵冶三期技改，形成年产40万吨铜生产能力，全年销售收入突破100亿元，成为江西工业首家销售收入过百亿元的企业。天施康药业、三川水表、南方照明、铜材及眼镜加工等企业退城进园，一批重大技术改造项目竣工投产，经济效益明显提高。非公有制经济加快发展，企业注册资金超过16亿元，从业人员达926万人，全年上交税收8600余万元。国有集体企业改制工作继续稳步推进，化解了一些历史遗留问题，部分改制企业焕发生机。工业园区建设取得重大进展。市工业园、贵溪工业园和余江工业园基础设施建设初具规模，管理体制进一步理顺，赋予了更多的服务职能。全市工业园区引进企业175家。电力建设明显加快。龙虎山110千伏、罗河110千伏输变电工程完工运营；贵溪电厂二期投资268亿元的两台30万千瓦机组已开工建设。

(二)积极推进农业产业化经营，农村经济发展明显加快

农业生产克服了特大干旱造成的严重影响，主要农产品产量稳中有增，粮食、油料、花生、水产品产量与去年基本持平，水果、肉类产量同比分别增长09％、37％。多种经营占农业总产值的比重达到74％，比上年提高3个百分点。大力推广农业科技，农产品优质品率进一步提高。农业产业化步伐加快，45家龙头企业实现销售收入644亿元，增长25％，带动基地面积60万亩，参与农户12万户。农产品流通组织化程度提高，成立了生猪合作社、农民企业家协会，成功举办了鹰潭市首届农产品展示展销会。农业基础设施不断完善，实施了中潢圩堤、白塔渠等重点工程，完成了硬石岭水库、塘湾水库和一批小型水库除险加固工程。第一期“国家农村饮水解困工程”通过验收，第二期工程基本完工，解决了404万农村群众饮水困难。退耕还林、长防林和国家生态公益林建设顺利实施。农村小康示范村建设全面启动，小康建设特派员工作取得初步成效。农村税费配套改革进展顺利，农民负担明显减轻。获全省农业农村工作先进市称号。

(三)坚持突出精品意识，城市面貌明显改观

以规划为龙头，遵循民主、科学的原则，对《鹰潭城市总体规划》进行了高起点修编。贵溪市、余江县也对城市总规进行了相应修编。实施了一批重点工程。全市关注的龙虎山大道工程、白露涵洞拓宽工程、鹰东连接线及梅园信江大桥工程胜利竣工。完成了天洁大道、沿江大道B段、东湖景观一期、五洲路、湖东路、院里安居小区等项目建设，改造了胜利路、湖西路、鹰潭火车站站前广场。对城市部分主要街道进行了美化、亮化，对小街小巷进行了硬化。管道液化气、新汽车站等项目动工兴建。206国道改造、锦黄线公路改造和梨温高速公路贵溪、余江连接线竣工通车。贵溪、余江、龙虎山均有一批重点工程竣工。出台了一批城市管理规章制度，开展了“告别陋习，树立新风”活动。对城市车辆乱停乱放、广告宣传乱贴乱画、经营摊点占道为市等影响市容市貌的不文明行为进行了专项整治。理顺了城市交通管理体制，平稳实现了出租车辆换型。通过加强建设和管理，城市框架拉大，城市功能增强，城市面貌改观，城市品位提升。

(四)克服“非典”负面影响，以旅游业为龙头的第三产业明显回升

面对“非典”疫情严重冲击，我市出台了一系列政策和措施，搞活市场，扩大消费，促进了第三产业稳步健康发展。旅游工作排难而进，在上海、福州、杭州、厦门、香港等城市举办了龙虎山旅游大型推介会，开展了多种形式的促销活动，进一步拓展了旅游客源市场。从去年7月开始，迅速扭转了因非典冲击带来的被动局面。实施了正一观大桥、上清大桥、龚资线改造、龙虎山旅游度假区中心广场、龙虎山宾馆改造、上清宫一期扫尾工程、古越民俗文化村等一批基础设施和景点项目建设。进一步理顺了管理职能，单列了市旅游局，强化了旅游行业管理和旅游市场整顿，促进了旅游服务质量的提高。龙虎山景区接待国内游客76万人次，直接收入2600万元，保持了不降而略有增长的好势头。房地产业快速发展，商品房竣工面积3515万平方米，增长2167％，一批住宅小区竣工。市场建设力度加大，建成了五金综合批发市场、水产批发市场。财政收支平衡，金融运行平稳。全市金融机构各项存款余额8939亿元，贷款余额9155亿元，分别增长187％和147％。邮电、通讯、保险、餐饮等行业发展速度加快。

(五)千方百计加大投入，经济发展后劲明显增强

坚持以招商引资为主抓手。全年实际引进内资315亿元，同比增长1143％；实际引进外资2678万美元，增长972％。引进企业规模明显扩大。贵溪新世纪水泥三期扩建、浙江天洁铜材加工、江西耐乐铜业、达尔漫鞋业、鹰港国际大酒店等一批项目投资规模超亿元。引进工业项目明显增多。全市引进500万元以上项目中，工业项目引资额占593％，达121亿元。积极参加省政府在北京、上海、厦门、南昌、大连、香港等地举办的招商引资活动，成功主办了在福州、浙江等地的招商引资活动，以诚招商、以特招商、以商招商、全民安商的氛围开始形成。外贸、外经工作成绩喜人，全年外贸出口完成2078万美元，同比增长232％，增幅居全省各设区市之首。

坚持多渠道筹措建设资金。认真做好项目前期工作，积极向国家和省申报，争取了一批资金，全年落实国债资金133亿元，增长482％；依法规范经营城市土地，有效盘活国有资产，筹措了一批资金，市本级全年土地收益11亿元；以诚信为本，通过银行贷款，融通了一批资金。其中省工商银行一次性授信2亿元贷款额度，已到位7000万元，有力地支持了城

市基础设施建设。

(六)坚持科教兴市战略，各项社会事业明显进步

创建全国科技进步示范市、建设国家新材料产业化基地工作取得重大突破。申报省、国家级各类科技计划项目46项，认定省级区外高新技术企业4家。我市被列为全省首批制造业信息示范市。调整了农村中小学校网点布局，进一步规范了中小学校收费行为，改造校舍4万平方米。加强了基础教育和职业教育工作，强化了各级各类学校管理。一手抓“扫黄打非”和文化市场整治，一手抓繁荣先进文化，一批文艺作品获国家和省表彰。市影剧院全面改造后

重新投入使用。首部《鹰潭市志》正式出版。全民动员、严防死守，有效地堵住了“非典”病源输入。建立了公共卫生长效管理机制，公共卫生突发事件应急处置能力得到提高。贵溪市新型农村合作医疗试点工作进展平稳。市人民医院综合大楼即将开工建设。全民健身活动荣获全省运动会团体总分二等奖，竞技体育迈上新台阶。全面完成了市县乡三级机构改革，全市党政机构由改革前的209个精减为162个，行政编制由改革前3997名减少为3221名。公务员管理日趋完善，事业单位人事制度改革试点工作稳步推进，专业技术人才队伍不断壮大。建立了一批社区服务机构，科教、文体、法律、卫生“四进社区”活动取得新成效，提高了群众生活品位。结合省辖鹰潭市成立20周年活动，表彰了一批为我市发展作出突出贡献的荣誉市民。坚持开展国防教育，努力做好双拥工作，获全省双拥工作先进市称号。廉政建设和反腐败斗争深入开展，源头治腐和整治经济发展环境取得阶段性成果。开展了开门评议机关活动，部门和行业作风进一步好转。认真执行人大及其常委会的各项决议，定期向人大常委会报告工作，广泛听取和采纳人大代表、政协委员及各民主党派、工商联、无党派人士以及市民群众的意见和建议，政府决策民主化、科学化水平进一步提高。广电行业管理加强，产业化发展迈出了实质性步伐。审计、人防、兵役、档案等各项工作取得新进步。计生、环保、国土资源工作不断加强，夯实了可持续发展的基础。

(七)高度重视困难群众生活，社会保障体系明显完善

千方百计筹措资金，确保了全市离退休人员养老金和下岗职工基本生活费按时足额发放。社保基金征缴力度加大，超额完成了省政府下达的全年养老保险基金征缴任务。全市有29015人纳入低保范围，做到应保尽保，调高了城市居民最低生活保障标准，对特困低保对象给予用电、用水、入学、医疗等方面的减免优惠。就业和再就业工作进一步推进，健全了服务机构，增加了资金投入，落实了税收、社保补贴、小额担保贷款等就业和再就业优惠政策，举办了春、夏、冬季劳务招聘洽谈会和人才招聘会，全市城镇新增就业人员13520人，实现下岗失业人员再就业4320人，其中就业困难人员2000人，对外输出劳务145万人。优抚安置工作成效明显，社会救助力度加大。建立了农村特困群众社会救助体系，成立了慈善机构和一批民办福利机构，开展了“慈善一日捐”等多种形式的社会捐赠活动。进一步扶持了民族乡村发展。全年城镇居民人均可支配收入7014元，增长104%；农民人均纯收入2717元，增长64％。城乡居民储蓄存款余额5988亿元，增长147％。

(八)坚持依法治市，社会稳定形势明显好转

认真落实行政复议法，行政工作法制化步伐加快。大力开展“四五”普法教育，增强了广大干部和群众的法制意识。建立了维护稳定工作领导责任制，完善了基层维稳网络。加强信访工作，坚持领导干部接待日和专线值班制度，群众反映问题的渠道进一步畅通。妥善处理了一批历史遗留问题，及时化解了发展中出现的新问题。加强了隐蔽战线的斗争。加强了点、线、面相结合的立体防控网络建设，社会治安综合控制能力明显提高。坚持不懈地抓好社会治安重点整治工作，刑事案发率下降，社会治安状况稳步好转。坚持不懈地抓好安全生产工作，建立健全了各级安全生产监管机构，落实安全生产责任，开展安全生产专项整治，全年生产安全、交通安全和消防安全态势平稳，未发生重大事故，国家和人民群众生命财产安全得到保障。

各位代表，本届政府已任期届满。1999～2003年的五年，全市人民面对全国、全省加快发展的逼人态势和改革、开放、发展的大好机遇，克服了前进过程中一个又一个困难，破解了发展进程中一个又一个难题，圆满完成了五届人大一次会议确定的各项目标和任务。这五年，改革力度不断加大，开放步伐不断加快，经济发展不断加速，社会事业不断进步，人民生活水平不断提高。据统计，全市国内生产总值年均增长109％；财政总收入年均增长141％，其中地方财政收入年均增长113％；固定资产投资年均增长296％；社会消费品零售总额年均增长90%；城镇居民人均可支配收入年均增长85％，农民人均纯收入年均增长43％。

各位代表，省委第十一次党代会确立的江西在中部地区崛起的发展思路和奋斗目标，对鹰潭加快发展产生了巨大的推动作用。今天鹰潭的发展变化，是省委、省政府正确领导的结果，是市委率领全市百万人民团结奋斗的结果，是市人大依法监督和市政协民主监督、积极支持的结果，也是与历届市政府打下的良好基础和社会各界的关心、支持分不开的。在此，我谨代表市人民政府，向全市人民致以崇高的敬意!向驻鹰人民解放军指战员、武警官兵、中央和省驻鹰单位、各民主党派、工商联、无党派人士、各群众团体、离退休老同志，向所有关心支持鹰潭建设的同志们、朋友们，表示衷心的感谢!

过去的五年，政府工作虽然取得了一定的成绩，但在经济生活中还存在不少困难和问题，政府工作还有许多缺点和不足。主要是：经济总量小，财政基础薄弱；经济结构不够合理，深层次体制矛盾仍然存在；就业不够充分，部分城乡居民生活困难；农业产业化规模不大，农民增收难度较大；影响社会稳定的因素仍然存在；政府职能转变尚未完全适应市场经济的要求，工作作风有待进一步改进。对这些问题我们将高度重视，采取有力措施加以解决。

二、今后五年的总体目标和2004年主要工作安排

今后五年，是圆满完成“十五”计划、启动实施“十一五”计划的重要五年，是全面建设小康社会的关键五年，也是我们把握机遇，迎接挑战，力争跨越式发展，加速崛起的五年。今后五年政府工作的总体目标是：深化改革，体制性、机制性、观念性障碍不断破除；扩大开放，全方位、多层次、宽领域的大开放格局基本形成；统筹兼顾，工业化、城市化、农业产业化进程明显加快，旅游成为支柱产业；协调发展，全市综合经济实力更加增强，社会事业更加繁荣，城乡面貌更加改观，人民生活更加殷实，人与自然更加和谐。力争国内生产总值年均增长115％，财政总收入年均增长13％，地方财政收入年均增长12％，全社会固定资产投资年均增长15％，社会消费品零售总额年均增长12％，城镇居民人均可支配收入年均增长7％，农民人均纯收入年均增长45%，人口自然增长率控制在省下达目标以内，城镇登记失业率控制在38％以内。

五年看头年。2004年是新一届政府的开局之年，做好今年的工作，将为全面完成新一届政府的各项目标任务奠定扎实的基础。2004年经济和社会发展的总体要求是：坚持以邓小平理论和“三个代表”重要思想为指导，全面贯彻十六大和十六届三中全会精神，认真落实全面、协调、可持续的科学发展观，坚持正确的发展思路，进一步解放思想、与时俱进，深化改革、扩大开放，突出重点、真抓实干，促进经济持续快速协调健康发展和社会全面进步。主要预期目标是：国内生产总值9026亿元，增长141％，其中一产增长40％，二产增长206％，三产增长100％；财政总收入10亿元，增长158％，其中地方财政收入506亿元，增长13％；全社会固定资产投资380亿元，增长230％；社会消费品零售总额278亿元，增长120％；城镇居民人均可支配收入7520元，增长72％；农民人均纯收入2865元，增长5.4％；城镇登记失业率控制在39％以内；人口自然增长率控制在省下达目标以内。

(一)坚持扶优扶强，加快提升工业化水平

坚定不移地推进工业化进程。加速培育支柱产业，提升传统产业水平，有重点地发展高新技术产业，逐步走出一条具有鹰潭特色的新型工业化道路。全年实现工业增加值42亿元，比上年增长207％。继续倾力支持驻鹰企业加快发展。按照“服务、联合、配套、补位”的工作思路，主动跟进，以服务促进企业扩张，以联合实现双赢，以配套、补位谋求自身产业发展。继续大力支持江西铜业集团公司达产达标和发展铜产品深加工项目。积极配合贵溪电厂二期扩建工程，力争贵溪220千伏输变电工程开工。进一步加大对重点工业企业的扶持力度，一厂一策，及时帮助企业解决生产中的困难和问题，支持企业做大做强。围绕优化结构，培植支柱，提高效益，在铜材加工、医药化工、精密制造和食品加工等行业，建设一批重大工业项目，形成区域特色鲜明的支柱产业群。加大技改力度，用高新技术改造传统产业，促进中小企业加快发展。建立工业发展激励机制，切实抓好企业改制扫尾工作，加快企业资产变现步伐，妥善解决改制遗留问题。大力发展国有资本、集体资本和非公有资本参股的混合所有制经济，完善中小企业信用担保体系。

全力推进工业园区建设，打造更具优势的工业载体和招商平台。进一步调整和优化工业园区总体规划，培植主导产业，突出发展重点，加大特色园区建设力度。依托现有产业基础，延长产业链，形成独具特色的产业群，使其成为带动我市经济发展的重要支撑力量。提高入园企业质量，注重引进一批科技含量高、财政贡献率大、产业关联度广、资源消耗低、环境污染少、人力资源得到充分发挥的现代企业。加强园区道路、绿化、供水、供电等基础设施建设。进一步强化园区服务职能，切实提高园区服务水平。

(二)加强农业基础地位，促进农村经济稳步发展

把解决“三农”问题作为政府工作的重中之重，把增加农民收入作为经济工作的中心任务，始终重视农业的基础地位，始终重视严格保护耕地和提高粮食综合生产能力，始终重视增加农民特别是种粮农民的收入。继续调整调优农业和农产品结构。强力做优粮油、蔬菜、茶叶、葛、食用菌、畜产品6大类食品加工产业，使食品加工业产值比上年增长30％。大力做强10个龙头企业，使全市规模以上龙头企业的产值增长20％。着力做大10个优质农产品基地，扩大面积、提高品质。努力做响10个优质农产品品牌，提高产品竞争力和市场占有份额。加大畜牧业发展扶持力度。增加农村富余人员就地就业人数，提高农民收入。积极发展生态农业，实施“无公害食品行动计划”，推进“农村沼气富民工程”进程。精心建设以洪五湖为核心的10万亩苗木花卉园和贵溪白鹤湖生态旅游观光农业园两个精品园。苗木花卉园今年新

增8000亩。进一步抓好长防林建设，做好退耕还林工程后续工作，抓好公益林建设和森林生态效益试点工程建设，切实加强森林防火工作。继续实施中潢圩堤除险加固、白塔渠灌区挖潜配套改造项目，抓好硬石岭水库库区小流域治理和枫林湾水库等一批中小型水库的除险加固。继续加快东湖城市防洪堤建设和夏埠城市防洪工程立项工作。做好地质灾害防治工作。多渠道筹措资金，增加农业投入。加强农业科技储备和农业技术推广，提高农产品的产量和品质。加强农产品市场体系建设，扶持农村各类专业合作组织发展，促进农产品流通。积极稳妥地推进土地使用权的合理流转，坚决遏制乱占耕地现象。深化粮食流通体制改革，继续解决好粮食企业历史遗留问题。深化农村信用社改革。巩固农村税费改革成果，切实抓好农村小康建设，确保农民得到更多实惠。

(三)切实做旺旅游业，全面繁荣第三产业

按照“大旅游、大市场、大产业”的发展思路，适度集中和有效配置经济资源、行政资源和社会资源，创新机制，改进方法，打好道教文化旅游牌，进一步实现龙虎山景区与市区联动，道教文化与旅游联动，逐步把旅游业建成一大支柱产业。开展创建中国优秀旅游城市活动，完善城市旅游功能，强化市民旅游意识，营造城市旅游氛围。积极改进旅游服务，强化旅游质量管理。以争创全国历史文化名镇为契机，进一步完善上清古镇建设规划，将道教文化、旅游景点与古镇改造相结合，把上清镇规划建设成为道教文化旅游观光的中心区。不断创新龙虎山景区管理体制和运作机制，力争早日完成龙虎山旅游集团公司股份制改造。举办第九届龙虎山文化旅游节，强化宣传和营销，使鹰潭龙虎山在全国形成较高的知名度。完成龙虎山仙水岩景区精品、天鹅湖景点、贵溪市白鹤湖、耳口和冷水景点、余江洪五湖等为重点的一批景点开发建设。加大旅游基础设施建设力度，完成龚资线等一批旅游公路改造工程，抓好应大线旅游公路建设，全面改造和完善龙虎山景区的供电、供水和通信设施。创造条件开展工业游、农业生态观光游、市区一日游等新的旅游项目。积极提炼鹰潭龙虎山道教养生、道教音乐和道教餐饮文化。加大旅游商品开发力度，着手规划建设旅游商品街市。全年接待境内游客和实现国内旅游总收入均比上年增长25％；接待境外游客和创汇分别比上年增长181％和47％。龙虎山景区旅游人次达到100万，旅游直接收入3500万元。

积极引导和扶持非公有制经济加快发展。放宽市场准入，非公有制企业在投融资、税收、土地使用和对外贸易等方面，与其他企业享受同等待遇。支持非公有制中小企业的发展，鼓励有条件的企业做强做大。立足区位交通优势，统筹规划市场布局，规范市场管理，促进市场繁荣。加快发展信息、会计、咨询等服务行业，提高服务经济的综合水平。积极支持房地产、邮电、通讯、交通运输、社区服务等行业的发展，带动第三产业全面繁荣。

(四)继续着力打造精品，实现城市面貌大变样

围绕城市整体面貌实现大变样的目标，按照量力而行、尽力而为的原则，做旺高桥区、提高梅园区、完善老城区、谋划夏埠区。依据城市总体规划，重点完成水、电和绿地系统等专业规划编制工作。加快城市路网建设步伐。沿江大道湖西路至梅园信江大桥段全线贯通。完成高桥区51号、53号路和梅园区25号路西段建设，启动府前路延伸工程。抓紧高桥区已出让土地的开工建设。完成东湖景观二期工程、梅园大道改造以及四海路、站江路立面改造工程。继续完善胜利路、站前广场改造工程。实施环城东路至梅枫路的亮化工程，同时对小街小巷分期进行亮化。完成管道燃气一期工程、江南水厂新增日供水35万吨扩建工程和垃圾填埋场项目。加快污水处理厂建设步伐。启动市体育中心一期、文化休闲广场、沿江小区、旧城西区建设工程，开工建设320国道市区段拓宽工程。抓紧筹建一批停车场，有效缓解市区停车难。切实抓好已出台的各项管理规章的落实，不断提高规划管理、街景管理、交通管理、环卫管理、物业管理的水平，依法做好征地拆迁工作，把城市管理纳入法制化、规范化轨道。继续开展告别陋习、树立新风活动，增强市民维护城市形象的意识。认真做好环境保护工作，不断提高城乡环境质量。积极推进小城镇建设。重点抓好锦江国家级小城镇建设和上清、邓埠、潢溪、龙虎山、塘湾、志光等6个省级重点镇建设。加快县到乡(镇)和乡(镇)到行政村公路建设步伐，全市安排农村公路建设34779公里。积极争取鹰景高速公路、贵溪人闽战备公路尽早立项动工。做好路政管理和道路养护工作。

(五)加大对外开放力度，多渠道筹集建设资金

加快发展开放型经济，对接长珠闽、融入全球化。按照“专业招商、全民安商”的格局，整合招商资源，完善招商机制，改进招商办法，健全以专业招商为主，以商招商、网上招商、代理招商和责任招商等多种形式的招商网络。进一步优化投资环境，营造浓厚的亲商、安商、富商的氛围。切实抓好项目库建设。突出工业项目招商，工业项目引资额达到引资总额的45％以上。切实提高签约项目的履约率、资金到位率、项目开工率和当年投产率。大力发展对外贸易。培育增加10家有自营出口经营权的企业，全力推出1家企业加入到全省重点出口企业行列中。积极应对外贸出口退税体制改革，选择扶持一批国家鼓励支持的高附加值、有发展前景和拥有知识产权的产品，增加化肥、木雕、瘦肉型猪、生物制药等优势产品出口。全年实际引进内资40亿元，实际利用外资3500万美元，出口创汇2286万美元，分别比上年增长27％、307％和10％。

继续积极争取国家和省有关部门的支持。按照国家宏观政策导向，做好项目筛选工作，在基础设施建设、改善农村生产生活条件、公共卫生、生态建设等方面推出一批项目，认真及时做好衔接工作。依法严格规范土地市场管理，坚持一级垄断、二级放开、三级搞活的原则，提高土地利用效率。支持民间资金兴办工业企业，引导鹰潭籍在外人员返乡办厂兴业，给予等同于外来企业的优惠和待遇。继续大力争取商业银行和地方金融机构的支持，加大对优势企业、工业园区、基础设施的信贷投放力度，加大对中、小企业和下岗职工再就业工程支持力度。切实做好农户小额信贷工作，增强服务“三农”意识。加强金融监管，整治社会信用环境，倡导和培育诚实守信的社会风气，防范和化解金融风险。

(六)统筹兼顾协调发展，实现社会事业新突破

坚持科教兴市战略，全面提高科技创新能力和公民素质。加快我市高新技术产业化步伐，重点支持新材料、机电一体化、生物医药、精细化工、铜制品精深加工等项目，力争全市省级区外高新技术企业达到10家。加快对机械、化工、仪器、铜材加工、建材、制药等主导产业的高新技术改造，实现传统产业结构升级。稳步推进市属科研机构改革，鼓励有条件的科研机构通过股份制改造，吸纳社会资金转制为民营科技企业。进一步抓实抓好基础教育尤其是农村义务教育，不断推进素质教育。切实做好农村义务教育“一费制”工作，确保生均公用经费按期足额到位。加大农村中小学一、二类危房改造力度。大力发展职业技术教育、民办教育和高等教育，不断提高办学水平。大力实施“希望工程”，广泛开展社会助学，帮助家庭困难的学生完成学业。切实加强公共卫生体系建设，建立健全四级疾控疫情监测与报告信息网络系统。不断提高对突发公共卫生事件、紧急疫情的快速反应和处理能力。加强农村卫生工作，新型农村合作医疗试点工作取得实质性进展。大力开展全民公共卫生活动，力争跨入省级卫生城市行列。充分发挥文化、广播电视繁荣社会主义先进文化的主渠道作用，促进城乡居民文明素质普遍提高。举办鹰潭市第五届运动会，促进群众体育和竞技体育协调发展。全面启动事业单位人事制度改革，建立开放有序的人才市场体系，积极培养和引进一批高层次、高新技术和紧缺人才。改革和创新统计方法，组织开展好第一次经济普查工作，不断提高经济运行监测预警能力。继续坚持计划生育基本国策不动摇，促进人口与经济协调发展。

(七)大力促进就业和再就业，切实维护社会稳定

继续实施积极的就业政策，努力拓宽就业门路。切实落实税费减免、小额贷款、社会保险补贴等再就业优惠政策。采取购买工作岗位等措施，优先安排“4050”人员再就业。规范劳动力市场秩序和企业用工行为，保障劳动者合法权益。建立劳动力市场信息监测中心，形成覆盖全市的劳动力市场信息网络。加大就业指导、技能培训力度，提高劳动者创业和转移就业能力。引导和组织农民有序流动，全年劳务输出14万人次。妥善解决因城市建设、工业园建设征用而失地的农民的就业和生活问题。积极争取中央和省转移支付再就业补助资金，切实加强资金监管。通过努力，确保全年城镇新增从业人员14300人，下岗职工再就业3500人，其中“4050”人员就业1500人。

完善社会保障体系。继续推进城镇各类企业及其职工、城镇个体工商户纳入社会保险统筹范围。依法妥善处理改制企业职工养老保险问题，并将企业离退休人员逐步纳入社会化管理。继续完善城市居民最低生活保障制度和农村困难群众救助体系，积极发展社会救济、救助和社会福利等事业，提高社会保障水平。完善扶贫开发机制，做好农村扶贫和民族乡村经济发展工作。积极开展争创全国双拥模范城市活动，扎实做好拥军优属优抚安置工作。

继续推进依法治市进程。切实抓好村(居)民自治工作，坚持和完善职工代表大会和其他形式的企事业单位民主管理制度，切实保障人民群众依法行使民主权利。严格行政执法责任制、评议考核制和错案责任追究制，坚决制止有法不依、执法不严和滥用职权的行为。认真抓好“四五”普法工作，以提高各级领导干部和政府公务员法律素质为重点，大力推进全民法制意识和法律素质的提高。加强新时期国防教育，支持军队和国防建设。坚持打防结合、预防

为主的方针，加强社会治安综合治理工作。完善国道交通治安防控、卡口防控、“网络化”巡逻防控、社区警务防控、边际防控运作机制，保持严打整治的高压态势，进一步增强群众安全感。认真做好信访工作，积极化解人民内部矛盾。狠抓各项安全生产，确保人民群众的生命和财产安全。

(八)加快服务型政府建设，不断提高行政水平

用足用活用好中央实行的积极财政政策，充分发挥财税的宏观调控作用。坚持依法治税，应收尽收，坚决不收过头税的组织收入原则，积极培植税源财源。加强税法宣传，强化税源监控，深化征管改革，建立健全护税协税网络，在抓好重点税种、重点税源征管的同时，切实抓好地方税种、零星税收的征管，确保财政收入稳步增长。坚持“一要吃饭，二保稳定，三搞建设”的原则，科学安排财政支出，重点保障政权建设、科技教育、社会保障等重点领域资金需要。推进财政管理体制改革，完善市直部门预算制度，启动市直单位会计集中核算工作，开展国库集中支付试点工作。改革市对县(市、区)财政管理体制，增强县(市、区)财政自身扩张能力。

转变和规范政府职能，提高行政效率。按照政企分开和精简、统一、效能的原则，切实把政府经济管理职能转到主要为市场主体服务，创造良好发展环境上来。继续推进依法行政，严格按法定权限和程序行使权力、履行职责。继续削减行政审批事项，完善办证中心统一办理行政审批事项功能。坚持政务公开，努力建设一支素质高、能力强、作风好的公务员队伍。

强化公共决策职能，提高决策水平。加强政府工作的前瞻性、全局性和操作性，不断提高政府决策质量。坚持科学民主决策，广泛开展人民群众建议征集活动，建立健全规划、价格等听证会制度，保证政府决策符合最广大人民群众的根本利益和普遍意愿。

加强和改进工作作风，自觉接受各界监督。努力创建学习型政府、学习型机关，争做学习型干部。坚持执政为民，时刻牢记“两个务必”，大力倡导求真务实、真抓实干的作风，力戒形式主义、官僚主义和弄虚作假。深入推进反腐倡廉工作，切实纠正损害人民群众利益的不正之风。坚持从严治政，接受市人大的依法监督，接受市政协和各民主党派、工商联、无

党派人士和人民团体的民主监督，接受人民群众和社会舆论的监督，树立为民、务实、清廉的政府形象。

各位代表，我们已经踏上了全面建设小康社会、加快社会主义现代化建设新的伟大征程。我们面临着前所未有的机遇和挑战，肩负的任务光荣而艰巨。让我们紧密团结在以胡锦涛同志为总书记的党中央周围，高举邓小平理论伟大旗帜，全面贯彻“三个代表”重要思想，在省委、省政府和市委的正确领导下，解放思想，把握机遇，奋力拼搏，开拓创新，为加快鹰潭的经济社会发展、创造鹰潭更加美好的未来而努力奋斗!
